# Weight Gain, Weight Loss, and Type 2 Diabetes Risk: Evidence From the Atherosclerosis Risk in Communities (ARIC) Study

**DOI:** 10.1002/edm2.70040

**Published:** 2025-04-08

**Authors:** Samaneh Asgari, Soroush Masrouri, Davood Khalili, Mojtaba Lotfaliany, Farzad Hadaegh

**Affiliations:** ^1^ Prevention of Metabolic Disorders Research Center, Research Institute for Metabolic and Obesity Disorders, Research Institute for Endocrine Sciences Shahid Beheshti University of Medical Sciences Tehran Iran; ^2^ IMPACT, The Institute for Mental and Physical Health and Clinical Translation, School of Medicine, Barwon Health Deakin University Geelong Australia

**Keywords:** atherosclerosis risk in communities study, incident, type 2 diabetes, weight gain, weight loss

## Abstract

**Introduction:**

While type 2 diabetes (T2DM) has become a major health issue in the North American and Caribbean region, the effects of weight change on incident T2DM, conditional on either initial or attained weight, are poorly addressed. Therefore, we aimed to assess the impact of 3‐year weight change on incident T2DM over 6 years among US individuals.

**Methods:**

A total of 8377 participants aged 45–64 years (4601 women), free of T2DM or cancer at baseline from the Atherosclerosis Risk in Communities (ARIC) study were included. Weight measurements were taken at baseline (visit 1, 1987–89) and approximately 3 years later (visit 2, 1990–92). Participants were categorised based on their weight change ratio into ≥ 5% weight loss, stable (±5%), and ≥ 5% weight gain. Cox proportional hazards models, adjusting for known diabetes risk factors, were used to estimate hazard ratios (HRs) and 95% confidence intervals (CIs) of incident T2DM, with stable weight (±5%) as the reference category.

**Results:**

During a median follow‐up period of 6 years, participants were classified into three categories: 361 persons remained stable (±5%), 47 with ≥ 5% loss, and 135 with ≥ 5% gain.

In multivariable analysis, after adjustment with initial weight, ≥ 5% weight gain and loss were significantly associated with higher [HR (95% CI): 1.68 (1.36–2.06), *p*‐value < 0.0001] and lower [0.73 (0.53–1.00), *p*‐value = 0.05] risks of incident T2DM, respectively. When adjusted for attained weight, weight gain ≥ 5% remained a significant risk factor for T2DM [1.51 (1.21–1.88)]; however, weight loss ≥ 5% lost statistical significance [0.84 (0.60–1.17), *p*‐value = 0.31].

**Conclusions:**

We found a robust association between weight gain and incident T2DM; however, the beneficial impact of weight loss was significantly attenuated after considering the attained weight.

## Graphical abstract

The current study suggests that each 4.5 kg of weight gain during 3 years (1‐SD increase) increases the risk of developing type 2 diabetes (T2DM) by about 30% among American population. Moreover, weight gain ≥ 5% of baseline weight during 3 years increases the risk of developing T2DM by about 1.3 fold, even after considering attained weight.

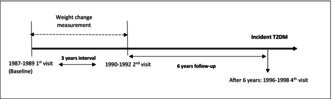

## 1 Introduction

Diabetes represents a major global health issue, with North America and the Caribbean (NAC) having the second‐highest prevalence of type 2 diabetes (T2DM) globally in 2021, affecting 14% of the population. The 10th edition of the International Diabetes Federation (IDF) report indicates that in 2021, the US had the highest diabetes prevalence within the NAC region, with a total of 32.2 million individuals affected [1].

Weight fluctuations, apart from obesity and overweight, have been linked to an increased risk of developing diabetes [2, 3, 4, 5]. Accordingly, it has been shown that considerable weight gain is a well‐known risk factor for incident T2DM [6, 7, 8]. Conversely, intentional weight loss achieved through diet and exercise has been shown to protect against T2DM development, even in individuals with pre‐diabetes [9, 10]. The American Diabetes Association (ADA) recommends a weight reduction of 3%–7%, which can delay the progression to T2DM in people with pre‐diabetes [4].

Previously, in a pooled analysis of Atherosclerosis Risk in Communities (ARIC), Coronary Artery Risk Development in Young Adults (CARDIA), and the Framingham Heart Study (FHS) cohorts, it was revealed that compared to Blacks, White individuals have a higher incidence of T2DM over a 9‐year follow‐up period for each one‐unit increase in log body mass index (BMI)‐years. This association was observed in both younger and middle‐aged individuals, with a more significant effect noted in the younger age group [11]. However, the impact of weight loss and gain on future T2DM risk, independent of attained weight, across sexes and according to age, remains an important knowledge gap. Women often present with excess weight gain at diagnosis and participate more in interventions, but men achieve greater weight loss and metabolic improvements, especially with > 7% weight loss. Bariatric surgery benefits women the most, reducing T2DM risk by up to 84%, while men see better outcomes with lifestyle changes and visceral fat reduction [12, 13, 14]. Furthermore, the menopausal transition might influence the relationship between weight change and the risk of developing T2DM. The decline in oestrogen levels during menopause leads to the accumulation of abdominal fat in postmenopausal women [15, 16], which is an established risk factor for developing insulin resistance and T2DM. Additionally, menopause is associated with a decline in muscle mass and strength, known as sarcopenia [17], further contributing to metabolic dysfunction and an elevated risk of T2DM, even in adults without overweight or obesity [18, 19]. However, the interaction between menopausal status and the effects of weight change on T2DM risk remains less understood. Moreover, the BMI–T2DM association is stronger in younger adults (< 30 years) than in older adults (> 60 years), and the obesity–T2DM link weakens with age [20, 21]. Despite this, older adults often show greater reductions in weight and haemoglobin A1c (HbA1c) associated with lifestyle interventions, as seen in the UK National Health Service Diabetes Prevention Programme [22]. While lifestyle interventions are effective in older adults, as shown in studies like Da Qing and the DPP [23], findings from the PREVIEW study suggest that older adults benefit less in body composition and cardiometabolic health despite greater sustained weight loss [24]. These differences underscore the importance of considering age subgroups when assessing weight change and T2DM risk.

To address this, we designed the current study utilising data from the ARIC cohort to assess the relationship between weight changes over a 6‐year follow‐up period and the risk of T2DM among the US population. Additionally, we analysed whether this relationship varied among different demographic groups, including men and women, Black and White individuals, younger and middle‐aged adults, as well as according to initial weight status.

## 2 Methods

### 2.1 Study Design and Study Population

The ARIC study was initially designed to study the development of atherosclerosis and its clinical sequelae. It also aimed to examine changes in cardiovascular risk factors, healthcare, and disease patterns with race, sex, location, and time. The Cohort Component of ARIC was initiated in 1987, and four field centers located in Washington County, MD; Forsyth County, NC; Jackson, MS; and Minneapolis, MN, were involved in randomly selecting and recruiting a sample of approximately 4000 individuals aged 45–64 years from specific populations in their respective communities. Participants underwent thorough examinations, including collecting medical, social, and demographic information. Participants were recruited between 1987 and 1989 (1st visit, baseline visit), with follow‐up reevaluations occurring at 3‐year intervals: 1990–1992 (2nd visit), 1993–1995 (3rd visit), and 1996–1998 (4th visit) [25]. As shown in Figure S1, for the current study, measurements of 1987–1989 were set as the 1st visit, and 1990–1992 was considered as the 2nd visit to calculate the weight change status of participants. Later, participants were followed up for 6 years (1996–1998) for the incidence of T2DM.

Of the 14,932 individuals aged 45–64 years individuals with T2DM at first and second visits (*n* = 2329), those with a history of cancer at baseline (*n* = 728), missing data on weight measurements at the first and second cycles (*n* = 1061), or any examined baseline variables including education levels, smoking status, fasting plasma glucose (FPG), blood pressure, triglycerides (TG), and high‐density lipoprotein cholesterol (HDL‐C) were excluded (*n* = 1350). Finally, after removing those with missing information on FPG at follow‐up measurements (*n* = 1087), 8377 individuals (4601 women) were eligible for the current study with a median follow‐up of 6 years until 1996–1998 (4th visit), the time laboratory measurements were available for FPG (Figure S2).

### 2.2 Clinical and Laboratory Measurements

Demographic information, medication usage, past medical history, family history of diabetes (FH‐DM), smoking behaviours, and educational levels were obtained through validated questionnaires administered by interviewers during visits [25, 26]. Weight was recorded using a digital scale, with participants wearing minimal clothing and no shoes, and the scale was rounded to the nearest 100 g. Waist circumference (WC) was measured in centimetres at the level of the umbilicus. Hip circumference (HC) was measured at the maximum protrusion of the gluteal region in centimetres. Three systolic and diastolic blood pressure (SBP and DBP) measurements were taken with a random‐zero sphygmomanometer, and the last two measurements were averaged. HDL‐C and TG were measured using standardised enzymatic assays. A modified hexokinase‐glucose‐6‐phosphate dehydrogenase procedure measured the FPG levels [27]. At each study cycle, medication history was collected by self‐reporting medication intake over the last 2 weeks and by reviewing medications brought by participants to the exams.

### 2.3 Variable Definitions

#### 2.3.1 Clinical Variables

BMI was calculated as weight (in kg) divided by height squared (in meters). According to WHO standards, the waist‐to‐hip ratio (WHR) was considered elevated at ≥ 0.90 for men and ≥ 0.85 for women. Abdominal obesity was defined as a WC > 102 cm for men and > 88 cm for women [28] Hypertension was defined as SBP ≥ 130 mmHg or DBP ≥ 80 mmHg or the use of antihypertensive medications.

#### 2.3.2 Sociodemographic Variables

Education levels were divided into three groups: (1) Grade school or 0 years of education, (2) High school but no degree, and (3) High school graduate or higher. Participants were asked whether they currently smoke cigarettes or whether they had done so in the past, yielding three categories of never, former, and current smokers.

#### 2.3.3 Past Medical and Family History

FH‐DM was based on self‐report (paternal and maternal, living or deceased). Prevalent cardiovascular disease (CVD) was defined as a composite measure of coronary heart disease (CHD), heart failure (HF), and stroke.

#### 2.3.4 Laboratory‐Based Variables

The TG to HDL‐C ratio (TG/HDL‐C) was calculated by dividing TG by HDL‐C. Incident T2DM was defined as having fasting (≥ 8 h) glucose ≥ 126 mg/dL (≥ 7 mmol/L) or the use of antidiabetic medications at follow‐up measurements (third or fourth cycles).

#### 2.3.5 Postmenopausal Definition

A woman was classified as postmenopausal if she met any of the following criteria: (a) experienced at least 24 consecutive months without menstruation, (b) underwent bilateral oophorectomy, or (c) had a hysterectomy and was 55 years of age or older at the time of the interview [29].

#### 2.3.6 Weight Change Percentage

Relative weight change (%) was calculated as:

$$\;\frac{{\text{weight}}_{2\mathrm{nd}\;\text{visit}}-{\text{weight}}_{1\mathrm{st}\;\text{visit}}\;}{{\text{weight}}_{1\mathrm{st}\;\text{visit}}}\times 100$$

We decided to use weight rather than height due to the marginal changes in height over 3 years. BMI primarily reflects variations in weight, and using weight directly provides a more accurate representation of changes without the influence of height fluctuations.

#### 2.3.7 Weight Change Cutoff

Furthermore, the weight change percentage was categorised as (1) loss ≥ 5%; (2) ± 5% weight change (reference group: stable); (3) gain ≥ 5%; [4].

### 2.4 Statistical Analysis

Baseline characteristics across weight change categories are shown as mean (standard deviation: SD) for normally distributed continuous variables, median (interquartile range: IQR) for skewed variables, and number (%) for categorical variables. Comparisons across weight change categories were performed using Student's ANOVA or *χ* ^2^ tests as appropriate. Cox proportional hazard models were used to evaluate associations of weight change categories with the incidence of diabetes. The interval from participants' second visit (1990–1992) until they developed diabetes, lost to follow‐up, or administrative censoring at 31 September 1998 (whichever came first) was defined as survival time.

Three models were designed in the multivariable analysis with covariates chosen by the literature review [9, 30]. Model 1 included weight change groups, sex, age, and race; Model 2 was additionally adjusted for baseline FPG and initial weight; and Model 3 was further adjusted with baseline measurements of WC, TG/HDL‐C, FH‐DM, current smoking, hypertension, prevalent CVD, and educational levels. Finally, to be comparable with other studies, regardless of any significant interaction between weight change categories with sex, age (< 55 vs. ≥ 55 years), race, or abdominal obesity (defined by WC, WHR, or BMI), multivariable analyses were carried out in split files as well. To be able to capture a potential nonlinear association between weight change and incident diabetes, multivariable restricted cubic splines with 4 knots at the 5th, 25th, 75th, and 95th percentiles were used [31]. As part of a sensitivity analysis, we repeated all analyses for women stratified by menopausal status. We also repeated all analyses using 3% and 7% weight‐change cutoffs to examine whether different thresholds would affect the results. Statistical analysis was performed using Stata (version 17 SE), and *p*‐values ≤ 0.05 were considered statistically significant.

## 3 Results

### 3.1 Baseline Characteristics

Of the total population, 9.1% and 21.9% had ≥ 5% weight loss and ≥ 5% weight gain, respectively, and about 70% of the participants had a stable weight over the first 3 years of follow‐up. Baseline characteristics of study participants according to the weight change categories are presented in Table 1. Significant differences existed among weight change categories except for race, prevalent CVD, and antihypertensive medication use. Characteristics of the participants across weight change categories during the baseline and second cycle are shown in Table S1. As predicted, weight loss ≥ 5% was associated with reduced WC, SBP, and TG levels over 3 years; however, among individuals with weight gain ≥ 5%, the mentioned variables had an increasing trend in addition to the values of DBP and FPG levels. For those classified in the weight gain category, the mean increase in weight was 6.6 kg, while individuals in the weight loss category experienced a mean weight decrease of 7.1 kg. Out of a total of 8377 participants, 361, 47, and 135 individuals experienced incidents of T2DM within the stable, decreasing ≥ 5%, and increasing ≥ 5% groups, respectively, during the median 6 years (IQR: 5.9–6.1 years).

**TABLE 1:** Baseline characteristics (1987–1989) of study participants across weight change categories: The Atherosclerosis Risk in Communities study.

	Decreasing ≥ 5% (*n* = 759)	Stable ± 5% (*n* = 5777)	Increasing ≥ 5% (*n* = 1841)	*p*
Age, year	54.4 (5.6)	54.0 (5.6)	52.8 (5.5)	< 0.001
Gender, female	455 (9.9)	2914 (63.3)	1232 (26.8)	< 0.001
Race, white	615 (8.9)	4822 (69.4)	1510 (21.7)	0.12
Education levels, year				< 0.001
Grade school or 0 years of education	166 (21.9)	936 (16.2)	291 (15.8)	
High school, but no degree	329 (43.4)	2441 (42.3)	826 (44.9)	
High school graduate or higher	263 (34.7)	2394 (41.5)	721 (39.2)	
Weight, kg	79.2 (16.8)	77.1 (15.5)	74.4 (15.6)	< 0.001
BMI, kg/m^2^	28.3 (5.5)	26.9 (4.6)	26.6 (4.8)	< 0.001
WC, cm	98.4 (13.8)	95.2 (12.5)	93.6 (13.2)	< 0.001
Waist to hip ratio	0.93 (0.07)	0.92 (0.08)	0.90 (0.08)	< 0.001
SBP, mmHg	121.0 (17.8)	118.8 (16.6)	115.4 (16.5)	< 0.001
DBP, mmHg	73.7 (10.8)	73.0 (10.4)	71.3 (10.3)	< 0.001
FPG, mmol/L	5.5 (0.52)	5.4 (0.48)	5.3 (0.44)	< 0.001
TG, mmol/L	1.24 (0.9–1.8)	1.21 (0.9–1.7)	1.11 (0.8–1.6)	< 0.001
HDL‐C, mmol/L	1.36 (0.44)	1.35 (0.44)	1.41 (0.44)	< 0.001
Current smoker, yes	200 (26.3)	1136 (19.7)	478 (26.0)	< 0.001
FH‐DM, yes	244 (32.2)	1680 (29.1)	612 (33.2)	0.002
Prevalent CVD, yes	35 (4.7)	238 (4.2)	87 (4.8)	0.49
Hypertension medication, yes	199 (26.2)	1354 (23.4)	467 (25.4)	0.09

Abbreviations: BMI, body mass index; CVD, cardiovascular disease; DBP, diastolic blood pressure; FH‐DM, family history of diabetes; FPG, fasting plasma glucose; HDL‐C, high‐density lipoprotein cholesterol; SBP, systolic blood pressure; TG, triglycerides; WC, waist circumference.

**FIGURE 1:**
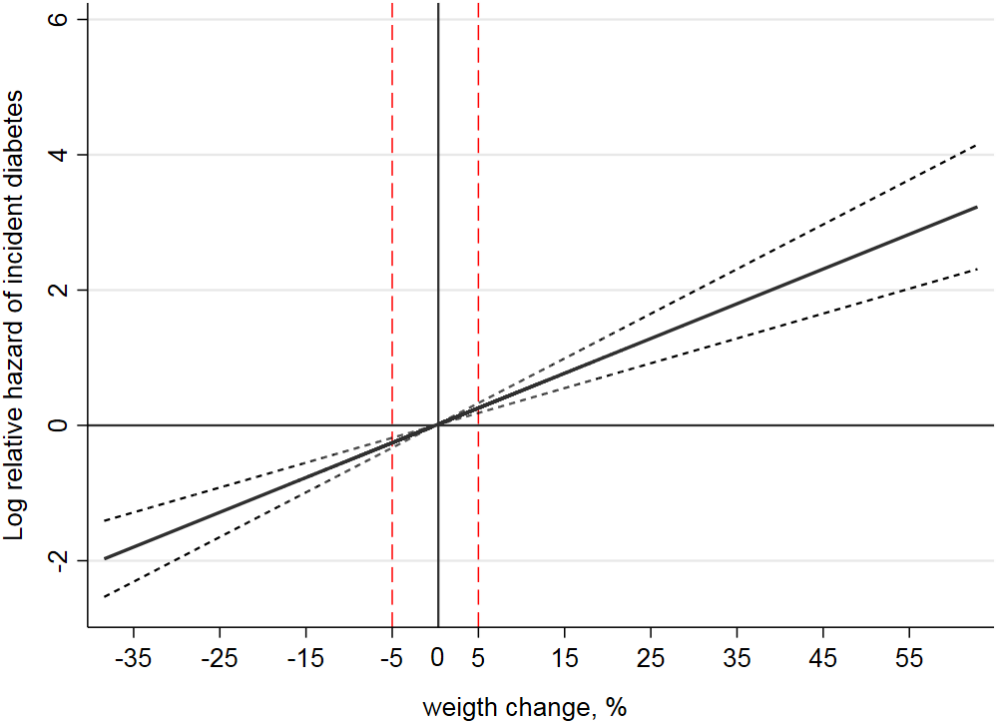
Restricted cubic spline curve for association (95% CI) of weight change with incident diabetes among the American population: Atherosclerosis Risk in Communities Study (ARIC).

Figure 1 shows the dose–response relationship between the risk of incident T2DM and weight change levels. A linear increasing relationship was observed between weight change levels and a 6‐year risk of incident T2DM.

### 3.2 Total Population Analysis

Table 2 illustrates the multivariable hazard ratios (HRs) and 95% confidence intervals (CIs), elucidating the association between categories of weight change (minimum 5%) and the occurrence of T2DM events. In comparison to individuals with stable weight (weight change within ±5% of baseline [i.e., from −5% to +5%]), after controlling for the potential confounding factors (model 3), increasing ≥ 5% was associated with a higher risk of incident T2DM for the 6‐year follow‐up [HRs (95% CI): 1.68 (1.36–2.06)]. Regarding decreasing ≥ 5% weight, compared with participants with stable weight change, after adjustment for age, sex, race, education levels, baseline measurements of FPG, initial weight, TG/HDL‐C, FH‐DM, current smoking, hypertension, and prevalent CVD (model 3), the HR (95% CI) for incident T2DM was 0.73 (0.53–1.00). Our data suggest that each 4.5 kg of weight gain during 3 years (1‐SD increase) increases the risk of developing T2DM by about 30% [1.30 (1.20–1.40)]. As presented in Table 2, we also adjusted the attained weight (at the second cycle) instead of the initial weight (at the first cycle) to investigate the importance of weight change categories at a certain attained weight level. The results revealed that the association of weight gain ≥ 5% with incident T2DM remained essentially unchanged [HR (95% CI): 1.51 (1.21–1.88)]; however, the association for weight loss ≥ 5% reached a non‐significant level [0.84 (0.60–1.17), *p*‐value = 0.31].

**TABLE 2:** Weight change association with incident diabetes through follow‐up: The Atherosclerosis Risk in Communities study.

	E/N	Adjustment for initial weight		Adjustment for attained weight	
		HR (95% CI)	*p*	HR (95% CI)	*p*
Model 1					
Stable ±5%	361/5777	Reference		Reference	
Decreasing ≥ 5%	47/759	0.98 (0.72–1.33)	0.91	0.98 (0.72–1.33)	0.91
Increasing ≥ 5%	135/1841	**1.29** (**1.05–1.57**)	**0.01**	**1.29** (**1.05–1.57**)	**0.01**
Model 2					
Stable ±5%	361/5777	Reference		Reference	
Decreasing ≥ 5%	47/759	0.79 (0.58–1.07)	0.12	1.00 (0.74–1.6)	0.98
Increasing ≥ 5%	135/1841	**1.67** (**1.36–2.05**)	**< 0.001**	**1.38** (**1.12–1.70**)	**0.002**
Model 3					
Stable ±5%	361/5777	Reference		Reference	
Decreasing ≥ 5%	47/759	**0.73** (**0.53–1.00**)	**0.05**	0.84 (0.60–1.17)	0.31
Increasing ≥ 5%	135/1841	**1.68** (**1.36–2.06**)	**< 0.001**	**1.51** (**1.21–1.88**)	**< 0.001**
1‐SD increase in weight change^a^, kg	543/8377	**1.30** (**1.20–1.40**)	**< 0.001**	**1.25** (**1.13–1.38**)	**< 0.001**

*Note:* Model 1: Weight change + age + sex + race. Model 2: model 1 + baseline fasting plasma glucose + initial/attained weight (as appropriate). Model 3: model 2 + waist circumference + triglycerides/high density lipoprotein cholesterol ratio + family history diabetes + current smoking + hypertension + prevalence of cardiovascular disease + educational levels. 1‐SD of weight change is equivalent to 4.5 kg.

Abbreviation: E/N, event/number.

^a^ Adjusted with covariates in model 3. Bold value are significant at *p* < 0.05.

### 3.3 Subgroup Analysis

**FIGURE 2:**
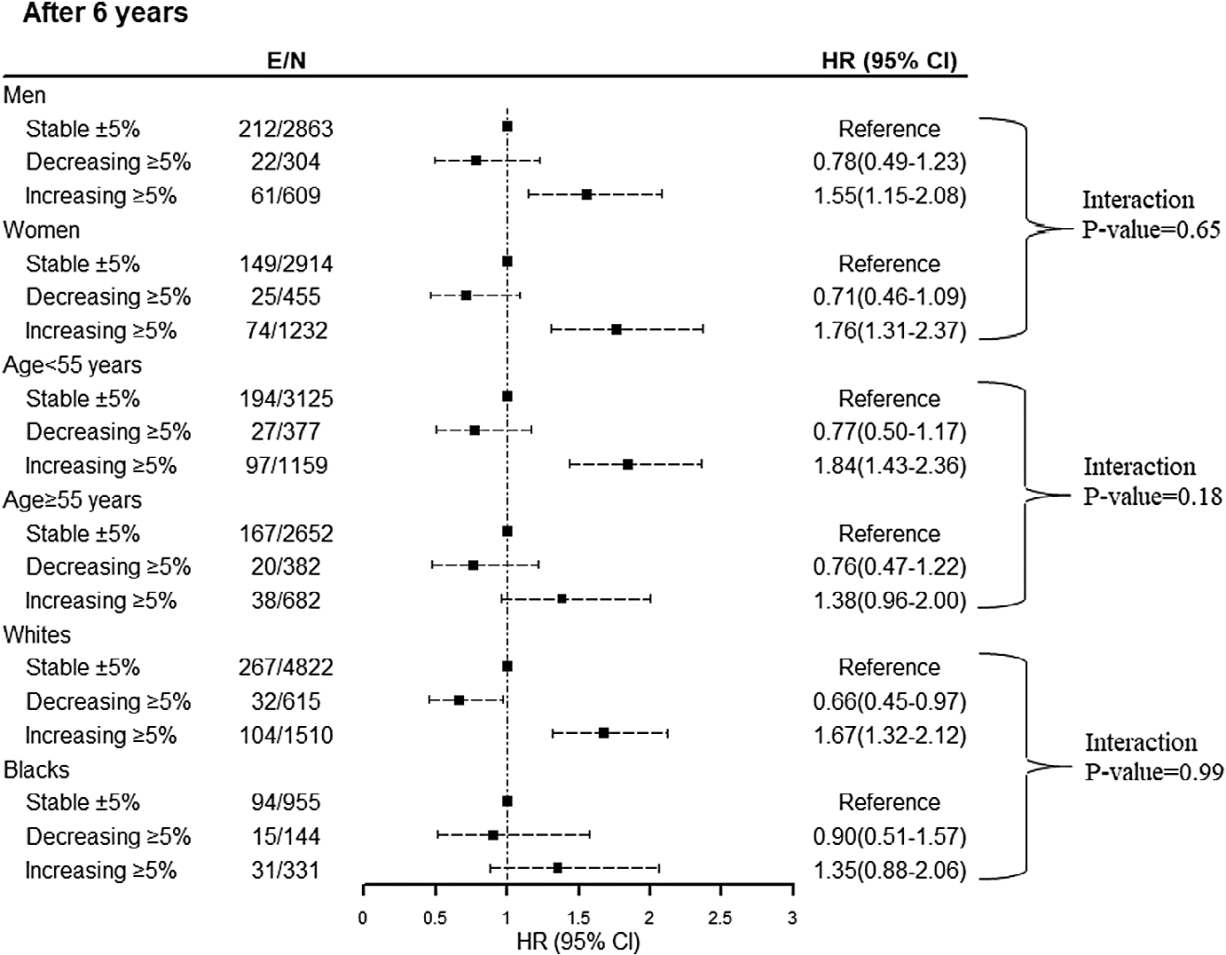
Weight change association with incident diabetes through follow‐up by sex, age, and race over 6‐year follow‐up: Atherosclerosis Risk in Communities Study (ARIC). Models are based on the Cox proportional hazard model and adjusted for weight change categories, baseline age, sex (except for sex‐specific), race (except for race‐specific), fasting plasma glucose, weight, waist circumference, triglycerides/high‐density lipoprotein cholesterol ratio, family history of diabetes, current smoking, hypertension, prevalence of cardiovascular disease, and educational levels. E, event, *N*, number.

**FIGURE 3:**
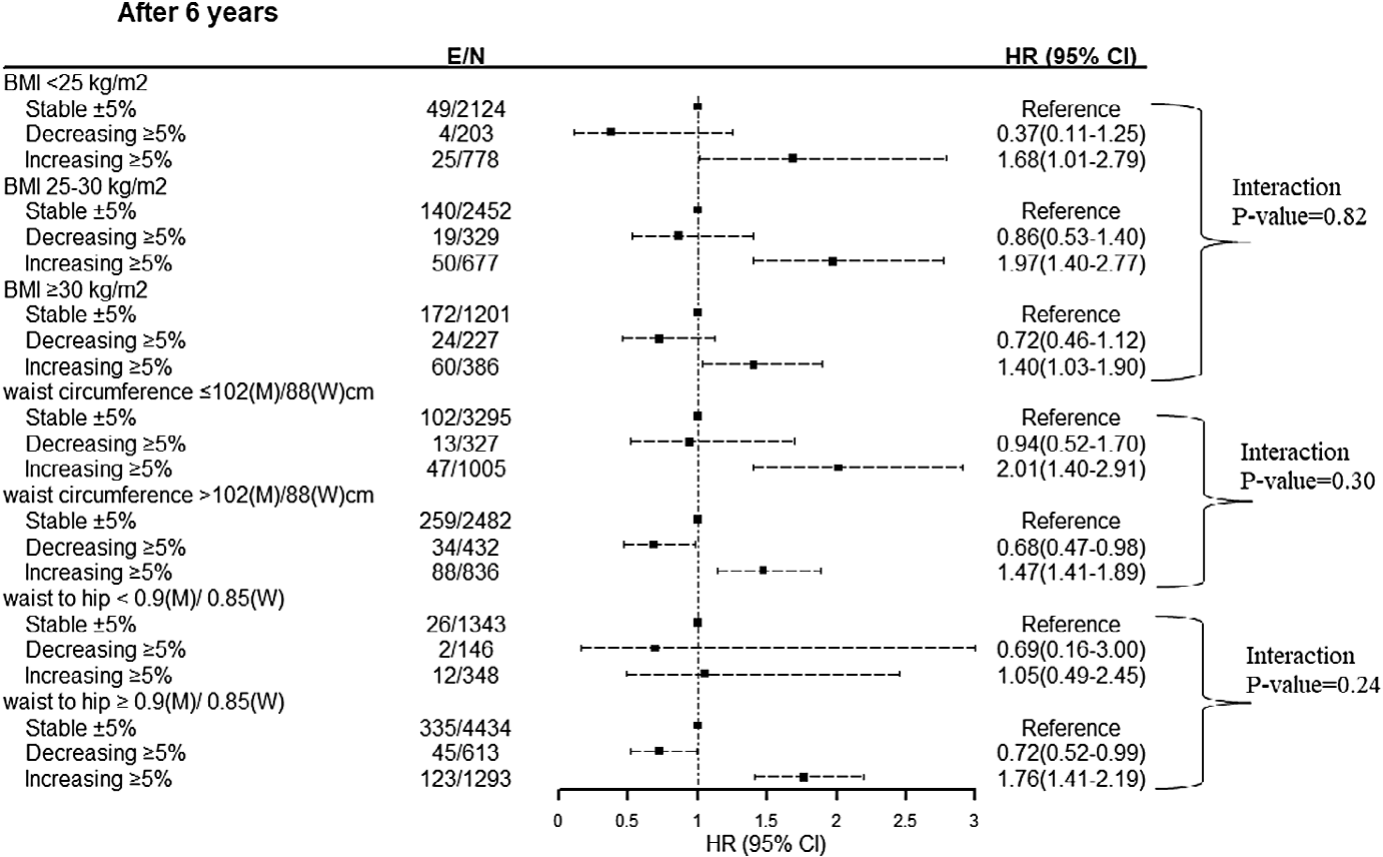
Weight change association with incident diabetes through follow‐up by BMI, waist circumference and waist to hip ratio categories over 6‐year follow‐up: Atherosclerosis Risk in Communities Study (ARIC). Models are based on the Cox proportional hazard model and adjusted for weight change categories, baseline age, sex, race, fasting plasma glucose, weight, waist circumference, triglycerides/high‐density lipoprotein cholesterol ratio, family history of diabetes, current smoking, hypertension, prevalence of cardiovascular disease, and educational levels. E, event, *N*, number.

To show the robustness of our findings, we examined whether the risk of 6‐year incident T2DM differed across subgroups of race (Whites vs. Blacks), age groups (≥ 55 years vs. < 55 years), sex (men vs. women), baseline BMI (< 25, 25–29, and ≥ 30 kg/m^2^), WC (≤ 102 cm in men/88 cm in women vs. > 102 cm in men/88 cm in women), and WHR (< 0.9 in men/0.85 in women vs. ≥ 0.9 in men/0.85 in women). As shown in Figure 2, no significant interactions of weight change with different subgroups were found, suggesting that the risk of incident T2DM as a function of weight change was similar among men and women, Whites and Blacks, those aged below and over 55 years, different BMI categories, WC categories, and WHR categories (*p* for interactions = 0.65, 0.99, 0.18, 0.82, 0.30, and 0.24 respectively) (Figure 3).

### 3.4 Women‐Specific Analysis

We also stratified all analyses by menopausal status in women (*p* for interaction = 0.56). We observed that the risk of developing incident T2DM across weight change categories was similar in both pre‐d postmenopausal women and was consistent with the total women results (Table S2).

### 3.5 Different Risk Change Analysis

In addition to the 5% change, we reanalyzed the data using 3% and 7% weight changes. We found that the results for the 7% change were consistent with those for the 5% change. Moreover, an increase of ≥ 3% in weight was associated with a higher risk of incident T2DM, while a decrease of ≥ 3% showed no significant association with incident diabetes (Table S3).

## 4 Discussion

This large‐scale and prospective study conducted among US adults revealed four main findings. First, a weight gain ≥ 5% was associated with a 68% higher risk of developing T2DM over a six‐year follow‐up period, even after accounting for the initial weight of participants as well as known diabetes risk factors. Second, a weight loss of ≥ 5% from baseline corresponded to a risk reduction of over 27% for incident T2DM during the same follow‐up duration. Third, the association between weight gain ≥ 5% and incident T2DM was resisted after adjustment for attained weight in place of initial weight; however, similar weight loss was not an independent predictor beyond its effect on attained weight. Fourth, in different subgroup analyses, we showed the robust association between weight change and the risk of incident T2DM.

Our findings extend previous work on the association between weight change and diabetes risk among US adults by demonstrating a significant association between relative weight gain and loss percentage of the risk of developing diabetes. in 2015, Wei et al. [11] published an article on three pooled cohorts of the US population. They compared adults aged 45–60 years, and each 1‐unit increase in log BMI‐years in adults aged 30–45 years significantly increased the risk of T2DM by 18% in Blacks and 35% in Whites over a 9‐year follow‐up. Considering weight change instead of changes in BMI, our study shows that each 4.5 kg weight gain over 3 years results in a 30% increase in the risk of developing T2DM during the subsequent six‐year follow‐up, which is aligned with the findings reported by previous studies [10, 32, 33, 34, 35, 36].

Based on the weight change ratio with adjustment for initial weight, our results suggest that weight gain ≥ 5% is associated with about a 70% rise in the risk of 6‐year incident T2DM, while weight loss ≥ 5% decreased the risk of developing T2DM during the same follow‐up period by about 30% in comparison with the stable weight change ratio group. In line with ours, Ohno et al. [10] research showed that an increase of ≥ 5% in BMI over 2.5 years, compared to a stable weight change group, was significantly associated with a 33% higher risk of developing T2DM among the Japanese population after adjustment for baseline BMI. In contrast, BMI loss over 5% was associated with an 18% reduction in the risk of incident T2DM.

In the current study, we found that achieving at least a 5% weight loss over 3 years can lower the risk of diabetes, independent of baseline weight. This finding aligns with ADA recommendations suggesting that individuals with pre‐diabetes, weight loss goal is over 5%–7% for reducing the risk of progression to T2DM [37, 38]. Similarly, among individuals with overweight or obesity and pre‐existing T2DM, a 5% weight loss can yield significant improvements in glycemic control [39, 40]. Despite this, the benefits of weight loss are shown to be incremental, and more substantial weight loss targets (e.g., 15%) may be appropriate for maximising clinical outcomes, based on individual needs, feasibility, and safety [41, 42]. In line with these findings, our study showed that weight loss of more than 7% was associated with a 44% lower diabetes risk, while a 5% reduction yielded a 27% significantly lower risk; in contrast, a 3% loss was non‐significantly associated with a 10% lower risk.

To ascertain the significance of weight change history at a specific attained weight level, we adjust the attained weight rather than the initial weight. Our findings revealed that weight gain ≥ 5% significantly impacts the incidence of T2DM, irrespective of attained weight measurements. This aligns with the study by Kaneto et al. [36] among a Japanese population aged 35–55, which found that weight gains of 6.0 to < 10.0 and ≥ 10.0 kg were significantly associated with about 2.30–3 times greater risk of diabetes, respectively, after accounting for attained weight. However, Jacobs‐van der Bruggen et al. [34] revealed that 5‐year weight gain was a significant predictor of diabetes independent of initial BMI but not independent of attained BMI among a Dutch population. Brancati et al. [43] also reported that a change in BMI 20–49 years among US men was not associated with diabetes risk independent of attained BMI. It has been shown that even modest weight gain leads to significant elevations in inflammatory markers [44] as well as basal plasma insulin, C‐peptide, and glucose levels [45], showing early activation of insulin resistance [46]. Moreover, it has been shown that enlarged subcutaneous abdominal adipocytes are associated with elevated diabetes risk [30, 47] and that individuals who experience weight gain, regardless of their attained weight, may have enlarged adipocytes, subsequently increasing diabetes risk independent of attained weight [48]. However, in our study, there was no evidence that weight loss was associated with a lower risk of incident T2DM after adjustment for attained weight, which was in line with previous studies among the Dutch and Japanese populations [34, 36]. Since only a few studies have adjusted attained weight, comparing these results with other studies in the field presents challenges.

Losing weight, achieved through lifestyle modifications or certain medications, is a key strategy for reducing the risk of incident diabetes [49, 50]. However, weight loss can be a double‐edged sword if not sustained [51]. Although studies indicate that lifestyle modifications, such as reducing calorie intake and increasing physical activity, offer the most promising path to sustained weight loss and lead to reduced diabetes incidence [38, 50], physical activity stands out as a more effective strategy for weight reduction and maintenance in overweight and obese adults; evidence shows that even without reaching specific weight goals, adequate physical activity significantly reduces the risk of incident diabetes [38].

Our measure of weight change did not distinguish between changes in lean and fat mass; previous research has shown that changes in total body fat mass rather than lean body mass predominantly drive the risk of T2DM, independent of baseline BMI or WC [52]. In our additional analyses, we did not observe statistically significant differences in the impact of weight change on T2DM risk across various WC or WHR categories, particularly regarding weight gain, suggesting that it per se remains an important determinant of T2DM regardless of baseline abdominal obesity. This aligns with findings from the Health Professionals Follow‐up Study [53], which showed that weight gain (both total and central) sharply increases T2DM risk. The importance of weight control for T2DM prevention is further underscored by the study among non‐obese Japanese individuals, where weight loss lowered diabetes incidence and weight gain raised it, despite the initial normal weight of study participants [54]. In a systematic review and meta‐analysis of 44 randomised and non‐randomised controlled trials, the protective effect of lifestyle interventions on T2DM risk did not differ significantly based on baseline obesity status [55]. Moreover, our study population consisted predominantly of individuals aged 45–64 years a group notably affected by sarcopenia. In a US study, the prevalence of sarcopenia among non‐diabetic adults was reported at 8.9% in the age group of 50–59 years and 14.6% in those aged 60–69 years [56]. Previous research has demonstrated that sarcopenia, independent of weight, can contribute to incident diabetes in overweight and obese individuals. Therefore, future investigations should explore the role of lean muscle mass in examining the impact of weight changes on diabetes risk [19].

Our subgroup analysis did not reveal any changes in the impact of sex, race, age, and BMI categories on incident diabetes. Likewise, a pooled cohort study conducted in Germany found no modification effect of sex on the incidence of T2DM [33]. Additionally, Ohno et al. [10], among a Japanese population, reported that ≥ 5% BMI gain as well as loss resulted in similar levels of increased and decreased risk of incident T2DM over 2.5 years, respectively, which were independent of individuals' weight categories (normal weight, overweight, and obese). We also did not observe any differences between pre‐ and postmenopausal women regarding the association between weight change and T2DM risk (i.e., in both categories weight gain about 5% was associated with more than 70% higher risk for incident T2DM). It has been shown that the menopausal transition leads to a greater accumulation of abdominal fat in postmenopausal women [15, 16], subsequently promoting insulin resistance and leading to T2DM. Additionally, menopause is associated with a decline in muscle mass and strength, known as sarcopenia [17], further contributing to metabolic dysfunction and an elevated risk of T2DM, even in adults without overweight/obesity [18, 19]. Our data analysis did not confirm the unfavourable impact of the menopausal transition on the development of T2DM compared with the premenopausal state.

Our study strengths include using objectively measured weight data in follow‐up visits, distinguishing us from several studies that rely on self‐reported data questionnaires and are susceptible to recall information bias. However, it is imperative to acknowledge certain limitations.

Firstly, although the ARIC study was initially designed to investigate atherosclerosis and cardiovascular outcomes, its features make it highly applicable to T2DM research [57, 58, 59]. The community‐based cohort, biracial population, large sample size, and long follow‐up period make it a valuable resource for studying T2DM risk within its demographic and longitudinal scope. Secondly, due to the absence of HbA1c and 2 h‐PCG data, diabetes was defined based on FPG; however, this is similar to the previously published articles on epidemiological studies [20, 60]. Thirdly, the measures of weight change do not differentiate between alterations in lean or fat mass, and finally, alcohol consumption was not available in the first visit, and therefore it was not considered in the analysis.

In conclusion, the current study suggests that weight gain ≥ 5% during 3 years increases the risk of developing T2DM by at least 30%, even after considering attained weight. However, we did not confirm the favourable impact of weight loss on incident T2DM independent of attained weight. Care should be considered regarding an individual's weight change; nutrition or lifestyle interventions are highly recommended to prevent future T2DM development [4].

## Author Contributions

Samaneh Asgari and Farzad Hadaegh conceived and planned the study. Samaneh Asgari and Farzad Hadaegh performed literature research. Samaneh Asgari conducted the analyses. Samaneh Asgari, Soroush Masrouri, and Farzad Hadaegh developed the first draft of the manuscript. Samaneh Asgari, Farzad Hadaegh, Davood Khalili, and Mojtaba Lotfaliany contributed to interpreting the data and critically revising the manuscript. All authors have read and approved the final manuscript.

## Ethics Statement

The Institutional Review Board (IRB) of the Research Institute for Endocrine Sciences (RIES), Shahid Beheshti University of Medical Sciences, Tehran, Iran, approved the secondary analysis of this study.

## Consent

The authors have nothing to report.

## Conflicts of Interest

The authors declare no conflicts of interest.

## Supporting information


**Data S1:** Supporting Information.
**Figure S1:** Timeline of the study design: Atherosclerosis Risk in Communities Study (ARIC).

## Data Availability

The datasets were obtained from the National Heart, Lung, and Blood Institute's Biologic Specimen and Data Repository Information Coordinating Center (BioLINCC) and are available in the case of the BioLINCC agreement.
