# Supplementary material for: Weight Gain, Weight Loss, and Type 2 Diabetes Risk: Evidence From the Atherosclerosis Risk in Communities (ARIC) Study
Source: Endocrinol Diabetes Metab. 2025 Apr 8;8(3):e70040. doi: 10.1002/edm2.70040 (PMC11978231; doi:10.1002/edm2.70040)
Supplement: Supplementary file 1 — Data S1: Supporting Information. Figure S1: Timeline of the study design: Atherosclerosis Risk in Communities Study (ARIC). [file EDM2-8-e70040-s001.docx]

| Table S1. Characteristics of study participants across weight change categories within first and second visit: The Atherosclerosis Risk in Communities (ARIC) study | | | | | | |
| --- | --- | --- | --- | --- | --- | --- |
|  | **Decreasing ≥5%**  **(n=759)** | | **Stable ±5%**  **(n=5777)** | | **Increasing ≥5%**  **(n=1841)** | |
|  | **1^st^ visist** | **2^nd^ visist** | **1^st^ visist** | **2^nd^ visist** | **1^st^ visist** | **2^nd^ visist** |
| Age, year | 54.4(5.6) | 57.3(5.6) | 54.0(5.6) | 56.9(5.6) | 52.8(5.5) | 55.8(5.5) |
| Gender, female | 455(9.9) | 455(9.9) | 2914(63.3) | 2914(63.3) | 1232(26.8) | 1232(26.8) |
| Race, white | 615(8.9) | 615(8.9) | 4822(69.4) | 4822(69.4) | 1510(21.7) | 1510(21.7) |
| Education level, year | | | | | | |
| - Grade school or 0 years of education | 166(21.9) | 166(21.9) | 936(16.2) | 936(16.2) | 291(15.8) | 291(15.8) |
| - High school, but no degree | 329(43.4) | 329(43.4) | 2441(42.3) | 2441(42.3) | 826(44.9) | 826(44.9) |
| - High school graduate or higher | 263(34.7) | 263(34.7) | 2394(41.5) | 2394(41.5) | 721(39.2) | 721(39.2) |
| Weight, kg | 79.2(16.8) | 72.1(15.0) | 77.1(15.5) | 77.6(15.6) | 74.4(15.6) | 81.0(16.9) |
| BMI, kg/m^2^ | 28.3(5.5) | 25.7(4.8) | 26.9(4.6) | 27.1(4.7) | 26.6(4.8) | 29.0(5.3) |
| WC, cm | 98.4(13.8) | 92.4(13.3) | 95.2(12.5) | 95.8(13.1) | 93.6(13.2) | 99.4(14.1) |
| SBP, mmHg | 121.0(17.8) | 116.6(18.2) | 118.8(16.6) | 119.3(17.2) | 115.4(16.5) | 120.1(17.3) |
| DBP, mmHg | 73.7(10.8) | 73.7(10.8) | 73.0(10.4) | 71.9(9.9) | 71.3(10.3) | 72.4(9.7) |
| FPG, mmol/L | 5.5(0.52) | 5.5(0.51) | 5.4(0.48) | 5.6(0.52) | 5.3(0.44) | 5.7(0.53) |
| TG, mmol/L | 1.24(0.9-1.8) | 1.10(0.8-1.5) | 1.21(0.9-1.7) | 1.24(0.9-1.7) | 1.11(0.8-1.6) | 1.32(0.9-1.9) |
| HDL-C, mmol/L | 1.36(0.44) | 1.39(0.45) | 1.35(0.44) | 1.29(0.43) | 1.41(0.44) | 1.31(0.43) |
| Current smoker, yes | 200(26.3) | 209(27.5) | 1136(19.7) | 1082(18.7) | 478(26.0) | 347(18.8) |
| FH-DM, yes | 244(32.2) | 244(32.2) | 1680(29.1) | 1680(29.1) | 612(33.2) | 612(33.2) |
| Prevalent CVD, yes | 35(4.7) | 35(4.7) | 238(4.2) | 238(4.2) | 87(4.8) | 87(4.8) |
| Hypertension medication, yes | 199(26.2) | 221(29.1) | 1354(23.4) | 1526(26.4) | 467(25.4) | 499(27.1) |
| BMI: body mass index; WC: waist circumference; SBP: systolic blood pressure; DBP: diastolic blood pressure; FPG: fasting plasma glucose; TG: triglycerides; HDL-C: high-density lipoprotein cholesterol; FH-DM: family history of diabetes; CVD; cardiovascular disease.  Note: data on follow-up measurements for education, FH-DM, and prevalent CVD were not available, therefore baseline values were replaced instead.  1^st^ visit (Baseline: 1987-89), 2^nd^ visit (1990-92) | | | | | | |

| Table S2. Weight change association with incident diabetes through follow-up according to the menopausal status in women: The Atherosclerosis Risk in Communities study | | | | | | |
| --- | --- | --- | --- | --- | --- | --- |
| Adjustment for initial weight | | | |  | **Adjustment for attained weight** | |
|  | **E/N** | **HR (95% CI)** | **p-value** |  | **HR (95% CI)** | **p-value** |
| Pre-menopause |  |  |  |  |  |  |
| - Stable ±5% | 79/1617 | Reference |  |  | Reference |  |
| - Decreasing ≥5% | 12/236 | 0.59(0.31-1.12) | 0.11 |  | 0.65(0.34-1.26) | 0.20 |
| - Increasing ≥5% | 53/811 | **1.81(1.26-2.60)** | **0.001** |  | **1.67(1.13-2.46)** | **0.01** |
|  |  |  |  |  |  |  |
| Post-menopause |  |  |  |  |  |  |
| - Stable ±5% | 70/1297 | Reference |  |  | Reference |  |
| - Decreasing ≥5% | 13/219 | 0.86(0.46-1.59) | 0.63 |  | 0.94(0.48-1.83) | 0.85 |
| - Increasing ≥5% | 21/421 | **1.72(1.01-2.94)** | **0.047** |  | 1.62(0.92-2.85) | 0.09 |
| Model 3: weight change + age+ race + baseline fasting plasma glucose + initial / attained weight (as appropriate)+ waist circumference + triglycerides / high density lipoprotein cholesterol ratio+ family history diabetes + current smoker + hypertension + prevalence cardiovascular disease + education levels  E/N: event/number | | | | | | |

| Table S3. Weight change association with incident diabetes through follow-up according to the different weight change categories: The Atherosclerosis Risk in Communities study | | | | | | |
| --- | --- | --- | --- | --- | --- | --- |
| Adjustment for initial weight | | | |  | **Adjustment for attained weight** | |
|  | **E/N** | **HR (95% CI)** | **p-value** |  | **HR (95% CI)** | **p-value** |
| Change percentage 3% | | | | | | |
| - Stable ±3% | 241/4046 | Reference |  |  | Reference |  |
|  | 81/1321 | 0.90(0.69-1.16) | 0.41 |  | 1.01(0.77-1.32) | 0.94 |
| - Increasing ≥3% | 221/3010 | **1.56(1.30-1.88)** | **<0.001** |  | **1.42(1.16-1.73)** | **0.001** |
|  |  |  |  |  |  |  |
| Change percentage 5% | | | | | | |
| - Stable ±5% | 361/5777 | Reference |  |  | Reference |  |
| - Decreasing ≥5% | 47/759 | **0.73(0.53-1.00)** | **0.05** |  | 0.84(0.60-1.17) | 0.31 |
| - Increasing ≥5% | 135/1841 | **1.68(1.36-2.06)** | **<0.001** |  | **1.51(1.21-1.88)** | **<0.001** |
|  |  |  |  |  |  |  |
| Change percentage 7% | | | | | | |
| - Stable ±7% | 436/6847 | Reference |  |  | Reference |  |
| - Decreasing ≥7% | 22/443 | **0.56(0.35-0.87)** | **0.01** |  | 0.66(0.41-1.05) | 0.08 |
| - Increasing ≥7% | 85/1087 | **1.72(1.36-2.19)** | **<0.001** |  | **1.41(1.17-1.94)** | **0.002** |
| Model adjusted for weight change + age+ sex+ race + baseline fasting plasma glucose + initial / attained weight (as appropriate)+ waist circumference + triglycerides / high density lipoprotein cholesterol ratio+ family history diabetes + current smoker + hypertension + prevalence cardiovascular disease + education levels  E/N: event/number | | | | | | |

| \| **6 years follow-up**  1987-1989 1^st^ visit (Baseline)  1990-1992 2^nd^ visit  Weight change measurement  **3 years interval**  After 6 years: 1996-1998 4^th^ visit  **Incident T2DM** \| \| --- \| |
| --- | --- |
| Figure S1. Timeline of the study design: Atherosclerosis Risk in Communities Study (ARIC) |

| 14,932 individuals 45-64 years of age participated in the 1^st^ visit (1987-1989) of ARIC study  Exclusion criteria:   - Those who experienced T2DM at the 1^st^ and 2^nd^ visits (n = 2,329) - History of cancer at 1^st^ visit (n=728) - Missing data on weight measurements at the 1^st^ and 2^nd^ visits (n=1,061) - Missing data on education levels, smoking status, FPG, TG, and HDL-C at 1^st^ visit (n=1,350) - Missing information on FPG at follow-up measurements (n=1,087)   Final Study participants 8,377 individuals (4,601 women) till 4^th^ visit |
| --- |
| Figure S2. Study flowchart  ARIC :Atherosclerosis Risk in Communities Study; FPG: fasting plasma glucose; TG: triglycerides ; HDL-C: high-density lipoprotein cholesterol |
